# Effective Combined Photodynamic Therapy with Lipid Platinum Chloride Nanoparticles Therapies of Oral Squamous Carcinoma Tumor Inhibition

**DOI:** 10.3390/jcm8122112

**Published:** 2019-12-02

**Authors:** Eka-Putra Gusti-Ngurah-Putu, Leaf Huang, Yih-Chih Hsu

**Affiliations:** 1 Graduate Program of Nanotechnology, Chung Yuan Christian University, Taoyuan 32023, Taiwan; rahekagusti@gmail.com; 2 Center for Commercialization of Cancer Theranostics, Chung Yuan Christian University, Taoyuan 32023, Taiwan; 3 Division of Pharmacoengineering and Molecular Pharmaceutics, Eshelman School of Pharmacy, University of North Carolina at Chapel Hill, Chapel Hill, NC 27599, USA; leafh@email.unc.edu; 4 Department of Bioscience Technology, Chung Yuan Christian University, Taoyuan 32023, Taiwan; 5 Center for Nanotechnology, Chung Yuan Christian University, Taoyuan 32023, Taiwan

**Keywords:** photodynamic therapy, lipid platinum chloride nanoparticles, drug delivery, combination therapy

## Abstract

Encapsulating cisplatin (CDDP) into liposomes to form lipid-platinum-chloride nanoparticles (LPC NPs) has shown a promising anticancer effect in melanoma, bladder, and liver cancer models. This promising anticancer effect of LPC NPs challenges us to study its implications in combination with photodynamic therapy (PDT). Herein, we report the therapeutic efficacy of PDT+LPC on a xenograft model of oral squamous cell carcinoma (OSCC). Results showed that PDT+LPC significantly reduced the tumor volume by up to ~112%. Meanwhile, LPC, PDT+CDDP, or the CDDP group showed ~98.8%, ~73.1%, or ~39.5% volume reductions, respectively. Histological examination suggests that PDT+LPC or LPC treatment showed minimal side effects on renal damage compared to either CDDP or the PDT+CDDP group. Immunohistochemistry staining (IHC) staining on Ki-67, CD31, cleaved caspase-3, TUNEL assays, and western blots of tumor suppressor p53 confirmed consistent results. Most importantly, PDT+LPC prolonged tumor growth inhibition, which leads to minimum chemotherapy treatment administrations. Results suggest that PDT cytotoxicity provided a potent additive effect towards chemotherapy efficacy. Therefore, combined PDT with LPC NPs enhanced the therapeutic outcome in human OSCC.

## 1. Introduction

Cisplatin is known to promote DNA interstrand crosslinks to hinder cellular processes such as replication, transcription, and up-regulation of the tumor suppressor gene of p53. The cytotoxicity of cisplatin triggers cell cycle arrest at the G2 phase, leading to tumor cell apoptosis and cell death [1,2]. Due to this potent cytotoxicity, cisplatin remains the frontline chemotherapeutic agent in various cancer types [3,4,5]. However, cisplatin also shows severe side effects including nephrotoxicity, diarrhea, vomiting and nausea, ototoxicity, neurotoxicity, and allergic reactions [1,3,5]. Limiting the dosage has been found to be beneficial in reducing the side effects, but this also restricts the therapeutic effect [4].

Nowadays, combining chemotherapy with another modality has been reported to be a promising option to enhance therapeutic outcomes [6,7,8]. Photodynamic therapy (PDT) has been reported to successfully cure patients with various types of cancers [9,10]. PDT destroys the solid tumor by using three major mechanisms: direct tumor-cell killing, vascular damage, and immune response [11]. PDT functions by utilizing a light-activated substance, a photosensitizer (PS), and an exposure of specific wavelengths of light and oxygen. The energy transfer cascades include two pathways known as: Type I (radicals and reactive oxygen species) and Type II (singlet oxygen) photochemical processes that eventually yield cytotoxic reactive oxygen species (ROS). In a biological environment, these toxic species can interact with cellular constituents, causing biochemical disruption to the cell and triggering both apoptotic and necrotic cell death [12,13]. PDT is also known to transiently increase blood vessel permeability, in turn increasing the nanoparticle uptake inside the tumor via a mechanism known as super-enhanced permeability and retention (SUPR) [14,15].

Despite its efficacy, PDT is limited by several factors such as tumor depth, tumor size, and tumor location [16,17]. To overcome this setback, in this study we aimed to combine PDT with LPC NPs by administrating LPC post PDT treatment. LPC NPs is one of several cisplatin formulations that involves utilizing liposome as the drug carrier [18,19]. A previous study showed the significant tumor inhibition of LPC in various cancer types [18,19,20]. Several unique features of LPC also have been reported, including a transient release up to 3–4 h with a sustained release of platinum and the neighboring effect properties [21]. The administration of PDT before LPC is purposely done to provide a potent cytotoxic environment and further enhance the therapeutic effect of chemotherapy. This is facilitated by the SUPR effect that occurs post PDT treatment [22]. In a previous study, we also showed that administration of PDT before gene silencing enhanced the treatment outcomes [23,24]. Herein, we are the first to find that combining photodynamic therapy (PDT) with lipid platinum chloride nanoparticle (LPC NPs) significantly enhanced treatment efficacy with minimal side effects of chemotherapy.

In this study, we aimed to investigate the therapeutic efficacy of PDT+LPC in inhibiting tumor growth in a xenograft OSCC mouse model. The cytotoxicity and SUPR effects triggered by PDT were expected to enhance the chemotherapeutic effect of LPC. The *in vivo* study results showed that PDT cytotoxicity serves as a potent additive effect towards LPC chemotherapeutic efficacy. Most importantly, it was found that the combined PDT+LPC prolonged the tumor growth inhibition, resulting in the minimal chemo drug administrations. Overall, a combination of PDT+LPC is an effective and a potential modality for cancer treatment.

## 2. Experimental Section

### 2.1. Materials

1,2-dioleoyl-*sn*-glycero-3-phosphate (sodium salt) (DOPA), 1,2-dioleoyl-3-trimethylammonium-propane (chloride salt) (DOTAP), and 1,2-distearoyl-*sn*-glycero-3-phosphoethanolamine-N-[methoxy(polyethylene glycol)-2000] (ammonium salt) (DSPE–PEG–2000) were obtained from Avanti Polar Lipids, Inc. (Alabaster, AL, USA). DSPE–PEG–aminoethyl anisamide (AEAA) was synthesized as reported previously [25]. Photosan-2 was sponsored from Seehof Laboratorium (Photosan-2, Wesselburenerkoog, Germany). Cisplatin or cis-Diammineplatinum(II) dichloride and other chemicals were purchased from Sigma-Aldrich (Cisplatin, St Louis, MO, USA).

### 2.2. PDT Light Source

PDT was carried out using a portable innovative LED Wonderlight light source (Chayah Therapeutics, Inc., Taipei, Taiwan) as previously reported [23,25]. Photosan-2 was used as the photosensitizer in this study. The light source had a high-power LED with a wavelength of 640 nm, a bandwidth of 20 nm, and a tunable power density of 1–400 mW/cm^2^. The light spot diameter was 1.8 cm at 1 cm of working distance. The light dose was set at 100 J/cm^2^ with a power density of 320 mW/cm^2^.

### 2.3. Formulation of LPC NPs

CDDP was encapsulated into LPC NPs as previously reported [18,19]. Briefly, CDDP was reversed to form CDDP precursor (cis-[Pt(NH_3_)_2_(H_2_O)_2_](NO_3_)_2_)) by causing a reaction of CDDP (0.20 mmol) with silver nitrate (AgNO_3 (aq)_) (0.39 mmol). Two distinct microemulsions of KCl_(aq)_ (800 mM, µL) and CDDP (200 mM, 100 µL) were mixed to synthesize back the CDDP. 1, 2-dioleoyl-*sn*-glycero-3-phosphate (DOPA) (34.6 mM, 185 µL) was used to form lipid platinum core NPs. The final LPC NPs, 1.0 mL of LP core, 100 μL of 20 mM DOTAP/cholesterol (molar ratio 1:1), and 50 μL of 20 mM DSPE–PEG–2000 or DSPE–PEG–AEAA were combined. Chloroform was evaporated and residual lipids were hydrated in 200 μL 5% glucose solution in order to form LCP NPs.

### 2.4. Characterization of LPC NPs

Particle size and zeta potential of LPC were determined using Malvern Zetasizer Nano series (Malvern, Westborough, MA, USA). TEM images of LPC were acquired using Bio-TEM Hitachi HT7700 (Hitachi, Chiyoda, Tokyo, Japan). Cisplatin concentration was determined using an inductively coupled plasma-mass spectrometer or ICP-MS 7500ce (Agilent, Santa Clara, CA, USA) and the drug loading was determined as previously explained [18]. Briefly, the final LPC was centrifuged at 12,500× *g* three times for 15 min each. Supernatant was removed, then the final pellet was resuspended and lyophilized for 8–12 h. Dried NPs were weighed and Pt content was determined using ICP-MS to calculate the amount of cisplatin. The drug loading of LPC was determined by the weight of cisplatin divided by the weight of purified NPs, as shown in Equation (1).

(1) $$Drug\;laoding\;\left(DL\right)=\frac{Amount\;of\;drug\;in\;NPs}{Total\;weight\;of\;drug\;}\times 100\%$$

### 2.5. Cell Culture

Oral cancer of Asian squamous cell carcinoma, SAS, was a gift from Prof. Liu, Yang Ming University. Cells were handled using the standard protocol for cell culture as previously reported [19,20]. Briefly, cells were growth using Dulbecco’s Modified Eagle Medium: Nutrient Mixture F-12 (DMEM/F-12) medium (Invitrogen, Grand Island, NY, USA) and supplemented with 10% fetal bovine serum (FBS) (Invitrogen) under humidity conditions of 37 °C and 5% CO_2 (g)_. Cells were cultured at 70% confluence and detached using 0.05% trypsin-EDTA (Invitrogen, Grand Island, NY, USA) before subculture in DMEM/F-12 medium.

### 2.6. Establishment of OSCC Xenograft Animal Model and Treatment Design

A Xenograft animal model was established [23,24] 160 µL of 5 × 10^6^ SAS cells in the presence of 200 µL matrigel (Corning, Bedford, MA, USA) and subcutaneously inoculated using a 28-gauge disposable needle at the lower right dorsal flank of a 7–9-week-old male BALB/cAnN.Cg-Foxn1^nu^ (National Laboratory Animal Center, Taipei, Taiwan, ROC) nude mouse. The xenografted mice were randomly separated into six groups (*n* = 5 per group) including (i) PBS (phosphate buffered saline), (ii) PDT, (iii) CDDP, (iv) PDT+CDDP, (v) LPC, and (vi) PDT+LPC. All of the treatments were administered via i.v. route administration. The CDDP or LPC NPs was given at dose of 3.0 mg/kg. Photosan was given at a dose of 2.0 mg/kg prepared by dissolving 0.05 mg photosan in 200 µL PBS for every 25 g weight of mouse according to previous studies [23,24]. The PDT procedure was performed for 11 min after 55 min administration of the Photosan-2 at 320 mW/cm^2^, 100 J/cm^2^, and 2 cm distance from the tumor surface. All treatments were performed at a tumor volume of 200.1 ± 3.5 mm^3^ (195–210 mm^3^). The tumor volume was determined as previously explained [23,24]. The animals were sacrificed on the 18th day. All excised specimens were fixed in 10% formalin for further uses. These studies were approved (approval number 104011) and carried out in strict accordance with the recommendations in the Guide for the Care and Use of the Institutional Animal Care and Use Committee of Chung Yuan Christian University, Chungli, Taoyuan, Taiwan. 

### 2.7. In Vitro Cell Viability

SAS cell viability tests were conducted in response to the toxicity of CDDP or LPC in a concentration-dependent manner (0, 0.2, 0.4, 0.8, 1, 1.5, 2, 2.5, 3, 3.5, 4, 4.5, 5, 5.5, 6, 6.5, 7, 7.5, 8, 8.5, 9, 9.5, 10, and 10.5 µg/mL). Thirty to forty thousand of SAS cells were seeded on 48-well plates followed by overnight incubation to reach 70% confluence. SAS cells were treated with CDDP or LPC for 24 h of treatment time. MTT assays were performed after 4 h of incubation.

### 2.8. Western Blot Analysis

Western blots were conducted as previously reported [23,24]. Briefly, 10–15 µg of protein samples were loaded into 5%/12% Bis-Tris acrylamide gels for electrophoresis. Samples were then transferred to PVDF membrane (Millipore, Billerica, MA, USA). Transferred membranes were blocked with BlockPRO^TM^ blocking buffer (Visual Protein Biotechnology, Taipei, Taiwan) followed by overnight incubation with rabbit primary antibodies against mouse mAb P53 (GTX28590, #1:250 dilution, GeneTex^TM^, Taipei, Taiwan, ROC) and P-P53_(ser 15)_ (9286, #1:1000 dilution, Cell Signaling, Boston, MA, USA), respectively. Secondary antibodies were performed using goat polyclonal anti-mouse IgG HRP-conjugate (SC-2005, #1:5000 dilution, Santa Cruz Biotechnology, Inc., Dallas, TX, USA) and then observed using enhanced chemiluminescence substrate (OPDO845, PerkinElmer, Boston, MA). GAPDH (GTX100118, #1:1000 dilution, GeneTex^TM^, Taipei, Taiwan) was used as an internal control followed by peroxidase-conjugated goat anti-rabbit IgG (GTX213110, #1:10,000 dilution, GeneTex^TM^, Taipei, Taiwan).

### 2.9. H&E and IHC Staining

Tissues were embedded in paraffin and sectioned followed by standard H&E staining protocol as previously described [23]. The antigen retrieval was started by deparaffinizing and hydrating of the tumor tissues section for IHC staining. Hydrogen peroxide incubation for 10 min was performed to inactivate the endogenous peroxidase. Tumor tissues were exposed with rabbit polyclonal anti-CD31 (ab28364, #1:100 dilution, Abcam, Cambridge, MA, USA), rabbit monoclonal anti-Ki-67 (ab16667, #1:200 dilution, Abcam, Cambridge, MA, USA), rabbit polyclonal anti-cleaved caspase-3 (9661, #1:200 dilution, Cell Signaling, Boston, MA, USA) by following the manufacturer’s instructions. DAB detection kit (Pierce, Rockland, IL, USA) was used for visualization. Olympus BX53F light microscope (Olympus, Shinjuku-ku, Tokyo, Japan) was used for histological examination. The quantification was done at ×40 magnification (5 images per group) using ImageJ software (National Institutes of Health, http://imagej.nih.gov/ij/). 

### 2.10. TUNEL Assay

Paraffin-embedded tumor tissue sections were deparaffinized, rehydrated, and pretreated with proteases. TUNEL assay was done using in situ Cell Death Detection Kit, POD (cat. no. 11 684 817 910, Roche, Mannheim, Germany), by following manufacturer’s instructions. The histological analysis was done using Olympus BX53F light microscope (Olympus, Shinjuku-ku, Tokyo, Japan). TUNEL-positive cells were counted at ×40 magnification (10 images per group) using ImageJ software (National Institutes of Health, http://imagej.nih.gov/ij/). 

### 2.11. In Vivo Toxicity Assay

Six- to eight-week-old C57BL/6JNarl mice (*n* = 3 per group) were tail vein injected with PBS, CDDP, and LPC at the same condition as for *in vivo* treatment protocol. Mice were anesthetized and sacrificed on the 18th day using a cardiac puncture technique. Collected blood was slowly transferred to a 1.5 mL eppendorf tube and clotted at an ambient temperature for 30 min, then separated at 1180× *g* for 10 min at 4 °C twice (Hermle Z233 MK-2; Hermle, Wehingen, Germany). The liver biomarkers including aspartate aminotransferase and alanine aminotransferase as well as kidney biomarkers such as creatinine, blood urea nitrogen, calcium, and phosphorus were analyzed from the collected serum. Cytokines concentrations of inflammatory factors such as IL-6, IL-12, and INF-γ were determined using an ELISA kit (MyBioSource, San Diego, CA, USA).

### 2.12. Statistical Analysis

Data presented as mean values ± SD. Statistical significance was determined using one-way ANOVA followed by Turkey’s method applied to raw data using SigmaPlot version 12.5 (Systat Software, San Jose, CA, USA), and the normality test was done using GraphPadPrism6 (GraphPad Software, San Diego, CA, USA) where *p* < 0.05 was considered to show significant differences.

## 3. Results

### 3.1. Characterization of LPC NPs

**Figure 1:**
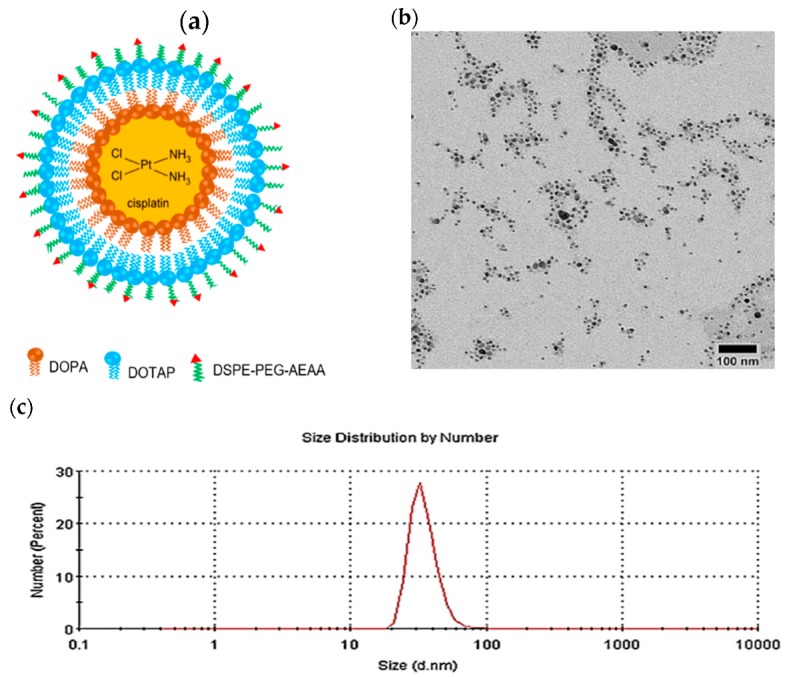
Characterization of lipid-platinum-chloride nanoparticles (LPC NPs). (**a**) Schematic illustration of the asymmetric bilayer LPC; (**b**) TEM image of LPC; and (**c**) size distribution of LPC measured using DLS. DOPA, 1,2-dioleoyl-*sn*-glycero-3-phosphate (sodium salt); DOTAP, 1,2-dioleoyl-3-trimethylammonium-propane (chloride salt); DSPE-PEG-AEAA, 1,2-dioctadecanoyl-*sn*-glycero-3-phosphoehanolamine conjugated with aminoethyl anisamide.

Cisplatin was successfully trapped and precipitated by utilizing the anionic lipid of dioleoylphosphatidic acid (DOPA) followed by outer leaflet coating of cationic lipid 1,2-dioleoyl-3-trimethylammonium-propane (chloride salt) (DOTAP) to form asymmetric lipid bilayer of LPC. 1,2-Dioctadecanoyl-*sn*-glycero-3-phosphoehanolamine conjugated with aminoethyl anisamide (DSPE–PEG–AEAA) was added as the outer leaflet of LPC NPs to target the overexpressed sigma receptor in a cancer cell (Figure 1a).

Transmission electron microscope (TEM) characterization revealed a fine homogeneous dispersion of LPC, as shown in Figure 1b. DLS measurement consistently confirmed the size distribution (Figure 1c). Results showed the mean LPC particle size was 35.0 ± 0.8 nm with a polydispersity index (PDI) of 0.4 ± 0.1 and a zeta potential value of 47.1 ± 0.4 mV as shown in Table 1. The drug loading was measured to be 89.6 ± 4.4% in different triplicate experiments.

### 3.2. LPC NPs Significantly Enhanced Oral Cancer Cell Death in Vitro

The *in vitro* anticancer efficacy of LPC was determined in the oral cancer of SAS cell lines. Either LPC or CDDP showed inhibition of cancer cell growth in a dose-dependent manner (Figure S1). The IC_10_, IC_30_, IC_50_, and IC_90_ of LPC NPs were 5.3 ± 0.4 µg/mL, 1.3 ± 0.4 µg/mL, 0.9 ± 0.1 µg/mL, and 0.2 ± 0.3 µg/mL, respectively, while the IC_10_, IC_30_, IC_50_, and IC_90_ of CDDP were 9.8 ± 0.4 µg/mL, 6.8 ± 0.4 µg/mL, 5.3 ± 0.4 µg/mL, and 0.3 ± 0.1 µg/mL, respectively. These results showed that in order to reach IC_50_, the LPC needs a 6.5-fold reduced concentration compared to CDDP (Figure S1). It suggests that LPC showed greater cancer cell killing effect than conventional CDDP. These results were obtained due to the small size of LPC, the capability to escape endosome-lysosome, and drug release at acidic condition in endosomes. A previous study showed that LPC NPs were highly endocytosed with minimum accumulation in lysosomes [18,21]. The endosomal/lysosomal escape capability of LPC was achieved *via* the “proton sponge effect” provided by DOTAP, a condition mediated by a positively charged agent with a high buffering capacity and the flexibility to swell when protonated during endocytosis [26,27,28,29]. As a result, an extensive inflow of ions and water into the endosomal environment occurred and subsequently led to the rupture of the endosomal membrane. This condition triggers the drug release from the encapsulation system [26].

### 3.3. In Vivo Therapeutic Outcome of Combined PDT and LPC NPs

**Figure 2:**
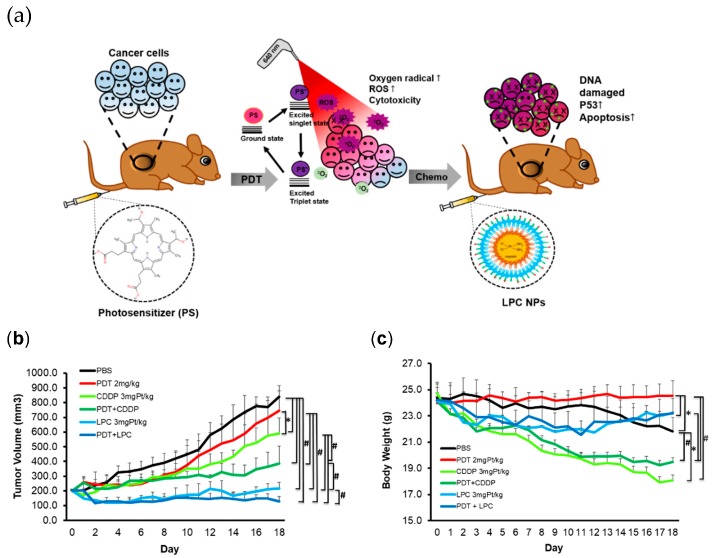
PDT combined LPC NPs prolonged the tumor inhibition *in vivo*. (**a**) Illustration of an *in vivo* study using PDT and LPC as a combination therapy (**b**) Tumor growth curve of treated SAS xenograft. (**c**) Body weight curve of treated mice. Data presented as mean ± SD, *n* = 5, * *p* < 0.01, ^#^ *p* < 0.001 compared either with the PBS group or with a combined PDT and LPC NPs group. PDT, photodynamic therapy.

Oral squamous cell carcinoma (OSCC) SAS tumor bearing xenograft models with 200.1 ± 3.5 mm^3^ tumor volume were randomly clustered into six different treatment groups including (i) PBS, (ii) PDT, (iii) CDDP, (iv) PDT+CDDP, (v) LPC and(vi) PDT+LPC. The PBS, CDDP, or LPC groups received three cycles of treatment with a 6-day interval. PDT was administered once both in PDT alone or combined therapy. In the combined therapy, either CDDP or LPC was administered twice during 1-day post PDT and 9-days after the first treatment. An illustration of the *in vivo* study can be seen in Figure 2a, and the treatment protocol is shown in the Table S1 Supplementary Table. 

The tumor volume of the PDT+LPC group significantly decreased (*p* < 0.001) with superior inhibition from 112.8% to 37.8% compared with the PBS or PDT group alone (Figure 2a). Compared to the PBS group, LPC NPs significantly reduced (*p* < 0.001) the tumor volume by up to ~98.8% or inhibited ~8.0% of tumor growth. Most importantly, we found that PDT+LPC or the LPC group alone significantly reduced (*p* < 0.001) the tumor volume by to 25.7% and 39.7% compared to the PDT+CDDP group, respectively. Combined PDT+LPC group significantly enhanced (*p* < 0.001) the therapeutic outcome in tumor volume reduction compared to that of PDT or LPC alone (~110.8% of tumor growth inhibition). Another critical finding was that PDT+LPC reduced the tumor growth rate, which led to fewer cycles of chemotherapy (CDDP or LPC). The PDT+LPC as the best treatment outcome required only two cycles of chemotherapy instead of three cycles (Figure 2b). These results dictate a promising application for clinical use involving better efficacy with minimal chemotherapy administration and fewer side effects. The PDT+LPC also significantly reduced (*p* < 0.001) side effects such as body weight reduction (Figure 2c) and toxicity (Table 2). In fact, the body weight of PDT+LPC and LPC groups increased by 5.3% and 4.7%, respectively. In contrast, massive weight loss of animals was observed in the CDDP group (*p* < 0.01) either alone or in combination with PDT (*p* < 0.001) compared to PBS by causing ~28.0% and ~20.2% weight loss reductions, respectively. Interestingly, PDT showed no body weight loss (*p* < 0.001) compared with PBS or LPC either alone or in combination. The PDT that maintained the body weight grew nearly 1.3% in contrast with PBS, which experienced weight loss roughly 11.5% due to the huge tumor sizes that developed.

**Figure 3:**
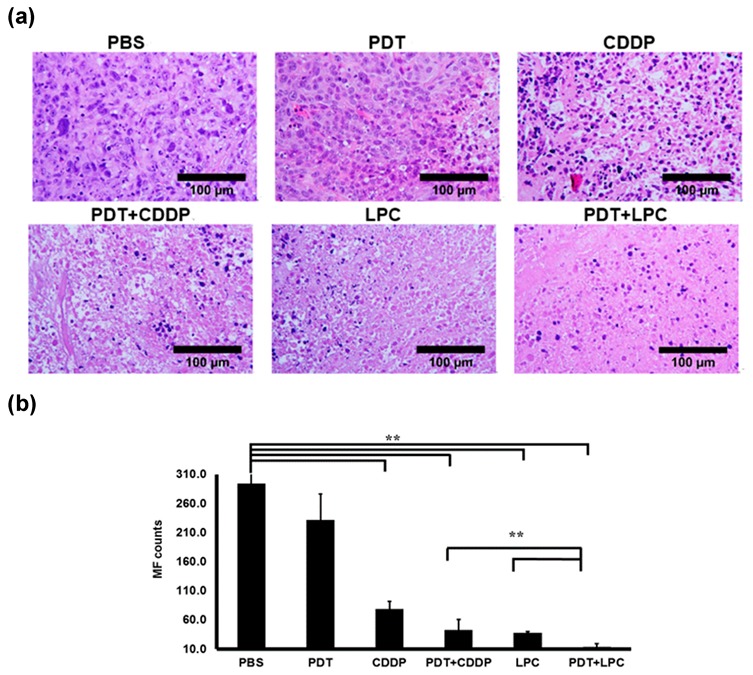
Hematoxylin and Eosin staining of tumor tissues. (**a**) H&E staining showed the tissue morphology of tumor treated mice. (**b**) quantification of mitotic Figure (MF) count for tumor tissues of each group. Results are shown as mean ± SD; scale bar means 100 µm, 40× magnification, ** *p* < 0.001, *n* = 5 for each. CDDP, Encapsulating cisplatin; PBS, phosphate buffered saline.

**Figure 4:**
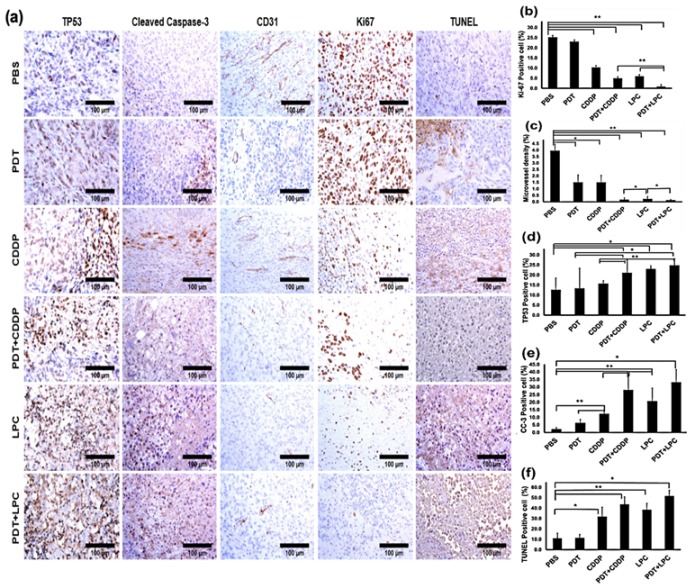
Immunohistochemistry staining. (**a**) a representative of tumor tissues IHC stained with tumor microvessel (CD31), cell proliferation (Ki-67), tumor suppressor gene of P53 total protein (TP53), apoptotic cell (cleaved caspase-3 and TUNEL assay), and quantification analysis of IHC staining of (**b**) Ki-67, (**c**) CD31, (**d**) TP53, (**e**) cleaved caspase-3, and (**f**) TUNEL assay. Results are shown as mean± SD; scale bar means 100 µm, 40× magnification, * *p* < 0.05, ** *p* < 0.001, *n* = 5 for each.

We further conducted histopathology assays of the excised tumor from each group, including hematoxylin and eosin (H&E) staining (Figure 3) and immunohistochemistry staining (IHC) in response to tumor suppressor protein p53 of total protein (TP53), tumor cell proliferation (Ki-67), tumor microvessel (CD31), and tumor cell apoptosis (cleaved caspase-3, TUNEL) (Figure 4). Based on H&E staining results, it was shown that the liver was not affected by PDT+LPC treatment but the kidney showed renal damage on either PDT+CDPP or the CDPP group. H&E staining of liver and kidney of each treatment group is shown in Figure S2 in the Supplementary Data. Abnormal tissue structures of the liver and renal damage such as tubular necrosis or loss of brush border were observed in a mouse treated with CDDP and combined PDT with CDDP (Figure S2). H&E also revealed a significant quantity of mitotic cells (*p* < 0.001) both in PBS and PDT groups (Figure 3b). As expected, PDT+LPC effectively reduced (*p* < 0.001) mitotic cells up to 15% while CDDP or PDT group showed the highest mitotic score of up to 27% and 79%, respectively.

The Ki-67 is a cellular marker indicating cell proliferation activity (Figure 4a). The positive Ki-67 cells revealed that PDT+LPC had the most potent anti-cancer killing effect (*p* < 0.001) at up to 3.2%, while LPC (19.2%), PDT+CDDP (23.3%) or CDDP alone (40.7%) was increased compared to the PBS group (Figure 4b). Taking together, H&E and Ki-67 staining collectively shared supportive results of PDT+LPC therapeutic outcomes in term of tumor volume reduction. To study each given treatment’s efficacy towards angiogenesis, we performed IHC using the microvessel positive marker CD31. Similarly, PDT+LPC significantly exhibited (*p* < 0.001) CD31 positive cell expression (Figure 4a). PDT+LPC significantly reduced (*p* < 0.001) the microvessel density (MVD) by up to 97% (Figure 4c). Meanwhile, PDT+CDDP, LPC, or CDDP groups were reduced by 96% (*p* < 0.001), 95% (*p* < 0.001), and 62% (*p* < 0.05) compared to PBS, respectively. In addition, PDT alone exhibited 65% (*p* < 0.05) of MVD, which was same as the CDDP group. These results suggest that PDT+LPC had the best performance results among all the groups.

The TP53 is a tumor suppressor protein involved in cisplatin cytotoxicity and IHC was performed on the TP53 marker to understand the impact of each given treatment on TP53 activation. TP53 expression was greatly increased in LPC treated groups either alone or in combination compared with PBS (Figure 4a). Both PDT+LPC and PDT+CDDP groups showed 96% and 67% of TP53 expression, or a 2.0- and 1.7-fold increase (*p* < 0.05) compared to the PBS group, respectively (Figure 4d). LPC alone significantly induced (*p* < 0.05) up to a 1.8-fold increase or 82% of TP53 expression compared to the PBS group. However, CDDP group only induced a 1.2-fold or 24% increase, while PDT alone group only had 6% of TP53 protein expression. These results indicated that the PDT+LPC treatment activated tumor suppressor gene p53 at the highest level in triggering the apoptosis pathway (Figure 4b).

The cleaved caspase-3 (CC-3) is an executor of cell apoptosis, and to reveal the role of CC-3 in our study we performed IHC against the CC-3 marker. CC-3 positive cells were found to be significantly increased in both combined PDT+LPC and PDT+CDDP (Figure 4a). Compared to the PBS group, PDT+LPC showed a 15.3-fold increase (*p* < 0.05), whereas PDT+CDDP showed a 13-fold increase (*p* < 0.001) (Figure 4e). CDDP or PDT alone showed only 5.7- and 2.9-fold positive apoptosis cell increases compared to PBS (*p* < 0.001). Meanwhile, LPC showed a 10-fold increase in cell death compared to PBS (*p* < 0.001) and a 3.2-fold increase compared to the CDDP group. This consistently suggests that PDT+LPC group showed the most prominent cancer cell killing efficacy.

**Figure 5:**
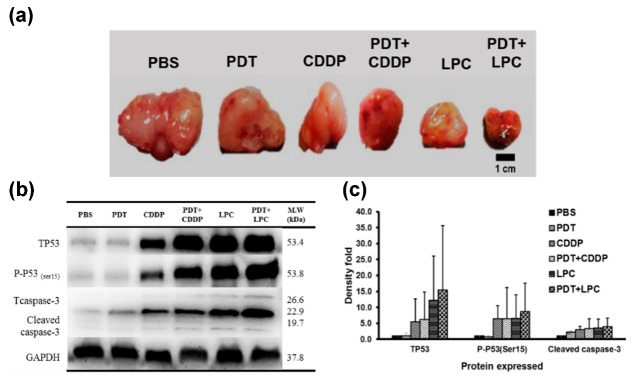
Western blot analysis. (**a**) excised tumor of each group; (**b**) protein expression post treatment in response to P53 (DNA damage), P53 phosphorylation of serine 15 (cell cycle arrest), cleaved caspase-3 (apoptosis) and GAPDH as a housekeeping protein for internal control; (**c**) the average quantification of protein expression from each group; Column indicates mean and bar indicates SD (*n* = 3).

To further elucidate the apoptosis features, we performed TUNEL assay to reveal the apoptotic DNA fragmentation on each group. As expected, the PDT+LPC group showed the most significant increase in the TUNEL positive cell (Figure 4a). Compared to the PBS group, there were 4.7- (*p* < 0.05) and 3.8-fold (*p* < 0.001) increases in PDT+LPC and PDT+CDDP groups, respectively (Figure 4f). Indeed, there was a 3.5-fold increase with LPC compared to the PBS group (*p* < 0.001) and a 2-fold increase compared to CDDP alone. Meanwhile, the CCDP alone only showed a 2.9-fold increase compared to PBS (*p* < 0.05). The PDT group did not significantly inhibit (*p* > 0.05) tumor growth and was consistent with the TUNEL positive cells with only 11.3 ± 3.5% cell death. Combined PDT+LPC showed the most apoptotic cells at up to 51.7 ± 5.7%, whereas PDT+CDDP and LPC showed 43.6 ± 7.2% and 38.5 ± 6.1%, respectively (Figure 4e). These results suggested the role of apoptosis in PDT+LPC group induced anticancer activity. Furthermore, the mechanistic studies also confirmed the greater anticancer effects of PDT+LPC or LPC alone (Figure 5).

Western blot assays were conducted using tumor lysates of each group against TP53 antibody (tumor suppressor protein) and p53 phosphorylation of serine 15 (P-P53_(ser 15)_). Results suggested that combined PDT with either CDDP or LPC NPs expressively enhanced both total p53 in response to DNA damage and P-P53_(ser 15)_ regarding cell cycle arrest (G2/M-phases) (Figure 5b,c). These were responsible for the tumor growth and explained the therapeutic outcome of each therapy. Most importantly, there was no expression of p53 phosphorylation of serine 20 (P-P53_(ser 20)_) in response to drug resistance. In addition, the EPR (enhanced permeability and retention) effect of LPC NPs was suspected to induce both considerably more DNA damage and cell cycle arrest than CDDP. Indeed, both PDT+LPC and PDT+CDDP displayed greater expression of P53 and P-P53_(ser 15)_ due to the capability of PDT to direct tumor-cell killing and trigger the immune response and vascular damage (the SUPR effect), allowing greater chance for the drug accumulation in tumor tissue. The neighboring effect promoted by LPC was also an adequate reason to explain this phenomenon. All results suggest that combined PDT+LPC promotes a superior anticancer effect, resulting in greater tumor growth inhibition where PDT served a potent additive effect, enhancing the chemotherapeutic efficacy of LPC NPs.

### 3.4. In Vivo Toxicity Assay

The toxicity of LPC was tested for both liver and kidney functions (Table 1). The C57BL/6JNarl mice were separated into three groups (i) PBS; (ii) CDDP; (iii) LPC following the treatment protocol of the *in vivo* study. 

Results showed that aspartate aminotransferase (AST) for the liver function was significantly different (*p* < 0.05) for CDDP compares to PBS and the LPC NPs group. This result is in line with the abnormality tissue structure observed in H&E staining of liver tissue (Figure S2). In contrast, the LPC NPs group did not show any significant difference to the PBS group (*p* > 0.05). Both CDDP and LPC NPs did not show a significant difference (*p* > 0.05) in alanine aminotransferase (ALT) for liver function compared to the PBS group. Although kidney function showed no significant difference for each group, the CDDP treated group showed the highest amount of blood urea nitrogen (BUN) and phosphorus levels. These biomarker elevations are caused by the nephrotoxicity effect of CDDP treatment [30]. Assayed concentrations of cytokines (IL-6, IL-12, and INF-γ) of three groups (PBS, CDDP, and LPC NPs) showed no significant activation of each biomarker in triplicates (*p* > 0.05) using IL-6, IL-12, and INF-γ, as shown in Table 1. 

## 4. Discussion

In this work, we attempted to evaluate the therapeutic outcome of combined PDT with chemotherapy on OSCC using a tumor-bearing mouse xenograft model. Although LPC NPs have shown great potential in tumor growth inhibition in various tumor types, no researchers have ever investigated the therapeutic effect of LPC in combination with PDT [18,19,20]. The PDT was firstly performed before LPC to provide a potent cytotoxicity enhancing the therapeutic effect of chemotherapy. In addition, PDT is known to transiently increase blood vessel permeability, thereby increasing the nanoparticle uptake inside the tumor [14,15]. A recent study showed that PDT triggers a phenomenon known as Super-enhanced permeability and retention (SUPR) and the enhanced permeability and retention (EPR) effect after PDT administration [22]. The SUPR mechanism was attributed to the formation of endothelial intercellular gaps as a result of endothelial cell microtubule depolymerization after PDT [15,22]. In a previous study, we have shown that administration of PDT before gene silencing of HIF1-A and VEGF-A using siRNA nanoparticle delivery enhanced treatment outcomes [23,24]. Henderson’s group have demonstrated this concept after a rapid uptake of nanoparticles inside a tumor *in vivo* [14]. A similar concept also has been applied to increase oncolytic virus accumulation inside a tumor *in vivo* [16]. Collectively, these rationales inspired us to study the combined therapy of PDT+LPC by firstly administrating the PDT. We believed that this study would provide a great contribution to the field, especially a comprehensive understanding on the best treatment strategy for pre-clinical use of LPC NPs.

Our study revealed that PDT cytotoxicity provided a potent additive effect by enhancing the LPC chemotherapeutic effect. This can be seen from our *in vivo* study that combined PDT+LPC treatment, which significantly inhibited tumor growth. Another study also showed that combined chemotherapy with PDT promoted significant cell-killing effects in cisplatin-resistant cells [31]. Similarly, a prominent cancer cell death was also reported in a study using cisplatin and pyrolipid combined with PDT [32]. Furthermore, Zhou et al. studied a combination therapy of SN-38 or 7-ethyl-10-hydroxycamptothecin, which is a chemotherapy drug with PDT, using graphene oxide as a drug delivery system. Their results showed that PDT + chemotherapy therapy exhibited antiproliferative effect on human lung adenocarcinoma cancer cell line A549 cells, in vitro [33]. A recent study using polymeric NPs of Doxorubicin and Ce6 photosensitizer as a chemo-PDT combination therapy also shared results suggesting that PDT + chemotherapy provided extraordinary treatment outcomes [34]. Kano et al also reported PDT and Doxil^®^ prolonged median tumor regrowth *in vivo* [22]. Altogether, our findings showed the promising performance of combined PDT + chemotherapy. The three mechanisms of PDT treatment in killing cancer cells including direct cytotoxic effects, vascular damage, and immune response induction may contribute to enhancing tumor growth inhibition. Since either LPC or CDDP were administered post-PDT, it may imply that the vasculature damage allowed a higher amount of chemo drug accumulation in the tumor, resulting in an enhanced chemotherapeutic effect of LPC [15,22]. These are also reasonable explanations for why PDT+CDDP enhanced tumor suppressor p53 protein expression of the excised tumor.

Siddik, Z. had noted some roles of tumor suppressor p53 in cisplatin cytotoxicity, including its activation as a direct consequence of DNA damage, a prerequisite for its function as a sequence-specific transcription activator, and its role for p53 in cisplatin-induced apoptosis [35]. Furthermore, Lahav’s lab [36,37] have intensively studied the dynamic behaviors of tumor suppressor p53. Their findings showed that p53 has an oscillatory dynamic [36], and cisplatin may upregulate both pro and anti-apoptotic pathways that act on two different apoptotic pathways on p53 activation [37]. They also found cells that acquire a large number of lesions might reach high p53 levels rapidly and enact apoptosis before inhibitors of apoptosis proteins accumulate. However, cells with fewer lesions will upregulate p53 slowly, leading to cell-cycle arrest and slow accumulation of apoptotic proteins [37,38]. Therefore, our findings on the upregulation of tumor suppressor p53 post cisplatin treatment either as a combination therapy or alone are relevant to these previous concepts.

*In vivo* study showed that CDDP affects both liver and kidney function either alone or in combination. An abnormal tissue structure of the liver and renal damage such as tubular necrosis or loss of brush border were observed in a mouse treated with CDDP. In addition, significant elevation in an AST serum lever of liver function was noted for the CDDP group. Although the kidney function showed no significant difference for each group, the CDDP treated group showed the highest amount of blood urea nitrogen (BUN) and phosphorus levels. These biomarker elevations are caused by the nephrotoxicity effect of CDDP treatment. It has been reported that elevation in serum phosphorus levels are associated with increased morbidity and mortality in renal dysfunction [30]. Furthermore, the CDDP group also showed the highest level of IL-6 and IL-12, which have been observed to be associated with cisplatin ototoxicity as well as with acute and chronic kidney disease [39,40]. 

## 5. Conclusions

In conclusion, our *in vivo* study does prove the concept of applying innovative LPC NPs (chemo drug) + PDT provided a significant impact towards clinical unmet needs. Results suggest that PDT cytotoxicity provided a potent additive effect towards chemotherapy. As for future directions, we are planning to incorporate the photosensitizer and chemo-drugs such as cisplatin, rapamycin, or gemcitabine in lipid-based nanoparticles for diagnosis and therapeutic purposes. Photosensitizers can be diagnostic agents and/or therapeutic agents. They could potentially become a novel theranostics approach for developing innovative combining treatment modality in the near future.

## Figures and Tables

**Table 1:** DLS characterization of LPC.

Run	Size (nm)	PDI	Zeta potential (mV)	%Drug Loading
1	34.94	0.48	47.40	89.32
2	34.22	0.48	47.10	90.92
3	35.81	0.41	46.70	88.51
Mean ^a^	35.0 ± 0.8	0.4 ± 0.1	47.1 ± 0.4	89.6 ± 4.4

^a^, mean ± standard deviation (*n* = 3). PDI, polydispersity index.

**Table 2:** Serum biochemistry and ELISA assay.

Group	AST (U/L)	ALT (U/L)	BUN (mg/dL)	Phosphorus (mg/dL)	Calcium (mg/dL)	IL-6 (pg/mL)	IL-12 (pg/mL)	INF-γ (pg/mL)
PBS	56.9 ± 6.8	23.3 ± 3.1	35.4 ± 1.6	4.9 ± 1.2	9.7 ± 0.3	650.7 ± 54.7	180.1 ± 24.9	100.2 ± 37.5
CDDP	124.7 ± 35.0 *	29.9 ± 7.2	40.5 ± 5.9	8.1 ± 2.3	10.5 ± 0.4	683.4 ± 81.6	240.6 ± 29.4	91.1 ± 32.5
LPC NPs	90.1 ± 46.4	30.7 ± 3.8	38.0 ± 4.5	5.9 ± 2.6	10.1 ± 0.9	603.7 ± 67.3	166.1 ± 12.9	90.7 ± 15.1

Data are given in mean ± SD, PBS, CDDP, and LPC NPs (*n* = 3). ALT (alanine aminotransferase); AST (aspartate aminotransferase); BUN (blood urea nitrogen); IL-6 (interleukin 6); IL-12 (interleukin 12); INF-γ (interferon gamma); PBS (phosphate buffered saline). * *p* < 0.05.

## Supplementary Materials

The following are available online at https://www.mdpi.com/2077-0383/8/12/2112/s1, Figure S1: LPC NPs induced cell death of OSCC SAS cell lines. Figure S2: H&E staining of liver and kidney tissue. Table S1: SAS xenograft model treatment protocols.
