# Supplementary material for: Effective Combined Photodynamic Therapy with Lipid Platinum Chloride Nanoparticles Therapies of Oral Squamous Carcinoma Tumor Inhibition
Source: J Clin Med. 2019 Dec 2;8(12):2112. doi: 10.3390/jcm8122112 (PMC6947167; doi:10.3390/jcm8122112)
Supplement: Supplementary file 1 [file jcm-08-02112-s001.pdf]

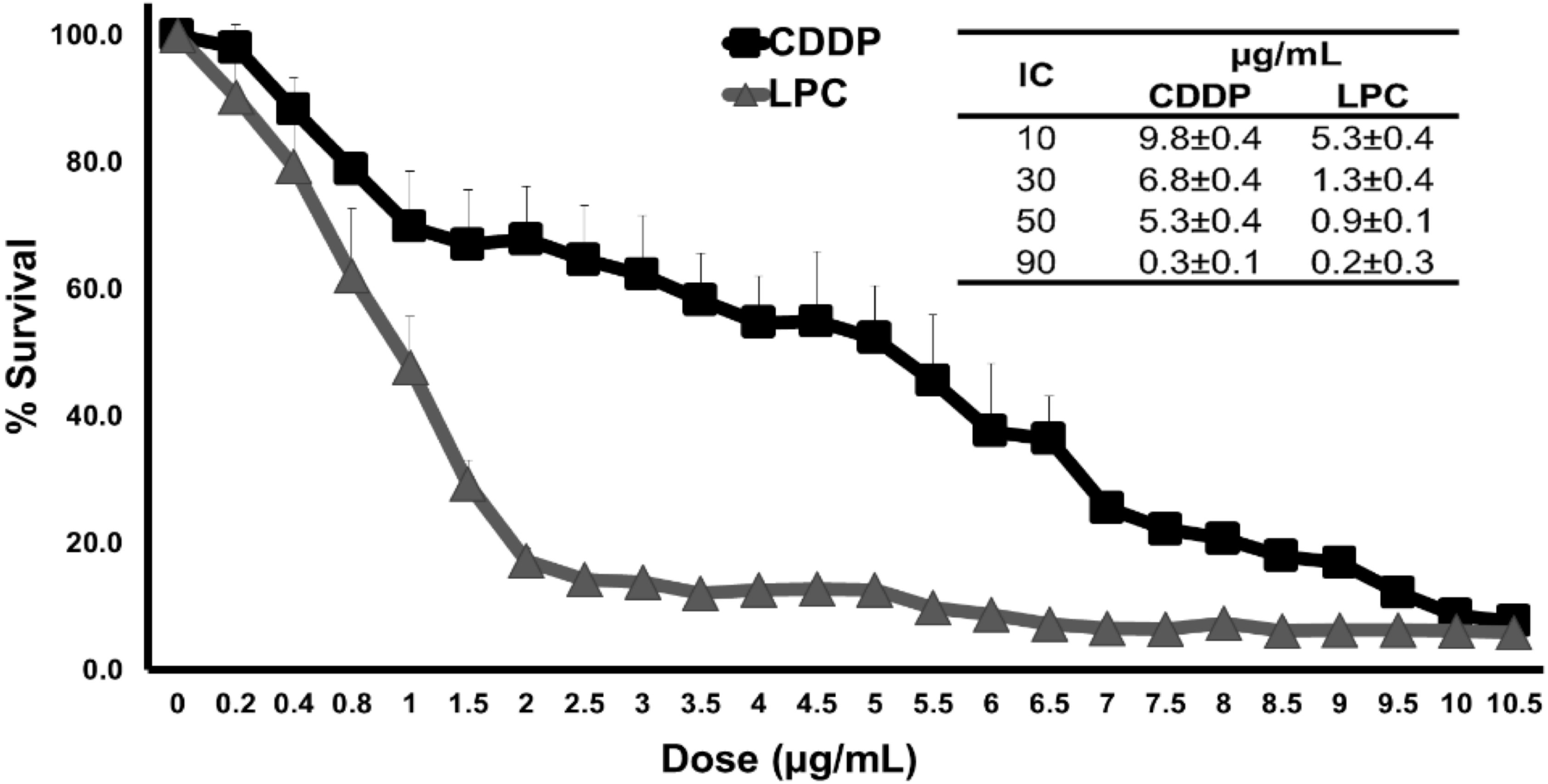

Figure S1. LPC NPs induced cell death of HNSCC SAS cell lines. The cytotoxicity of SAS cells following CDDP or LPC NPs treatment. LPC NPs treatment induced significant cell death in a dose-dependent manner. All groups were done in triplicate (N=3) of the individual experiment, the bar is shown as mean ± SD.

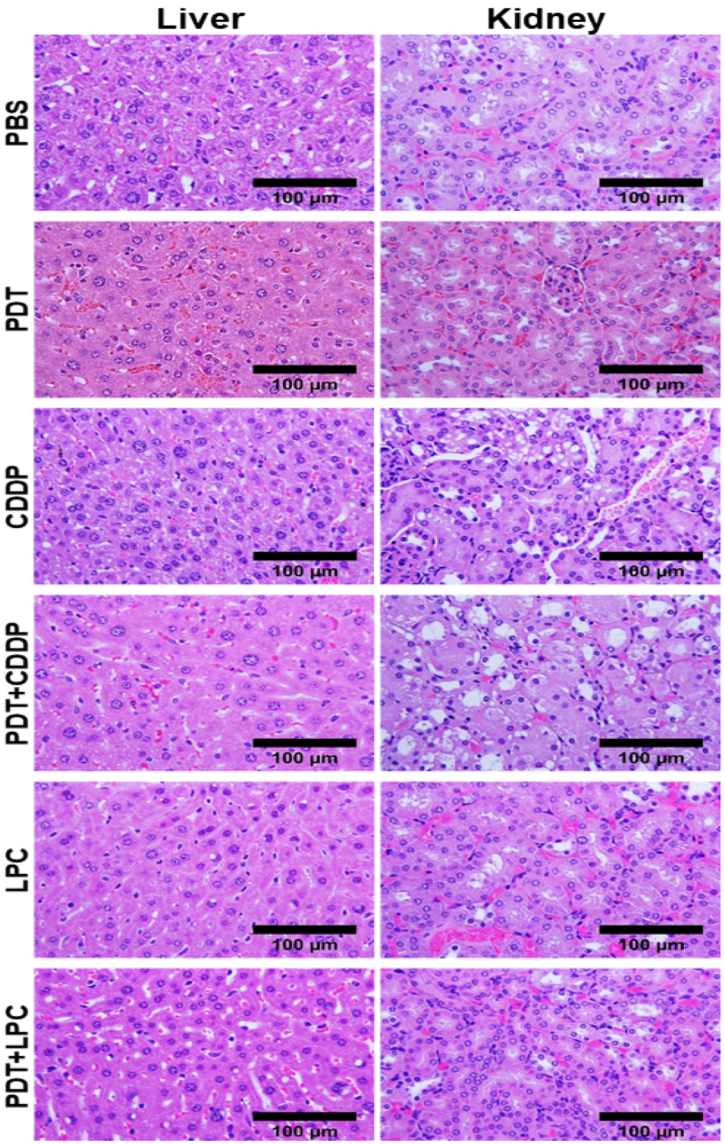

Figure S2. Hematoxylin and Eosin staining of liver and kidney. H&E staining showed the tissue morphology of liver and kidney of treated mice. Abnormal tissue structure of liver and renal damage such as tubular necrosis or loss of brush border were observed in a mouse treated with CDDP and combined PDT with CDDP.

Table S1. SAS xenograft model treatment protocol

Group	Treatment administration
**PBS**	Day 0, 6, 12
**PDT**	Day 0
**CDDP**	Day 0, 6, 12
**PDT+CDDP**	PDT on day 0<br>CDDP on day 1 and day 9
**LPC**	Day 0, 6, 12
**PDT+LPC**	PDT on day 0<br>LPC on day 1 and day 9
